# *Microbacterium chionoecetis* sp. nov. and *Agrococcus chionoecetis* sp. nov.: Novel Gut Bacteria from Red Snow Crab

**DOI:** 10.4014/jmb.2412.12044

**Published:** 2025-04-24

**Authors:** Dhiraj Kumar Chaudhary, Sang-Eon Kim, Hye-Jin Park, Kyoung-Ho Kim

**Affiliations:** 1 Department of Microbiology, Pukyong National University, Busan 48513, Republic of Korea; 2 Division of Marine and Fisheries Life Sciences, Pukyong National University, Busan 48513, Republic of Korea; 3 Korea Institute of Ocean Science and Technology, Busan, Republic of Korea

**Keywords:** *Microbacterium chionoecetis* sp. nov., *Agrococcus chionoecetis* sp. nov., red snow crab, gut microbiome, phylogeny

## Abstract

Two yellow-coloured, Gram-stain-positive, oxidase-negative, aerobic, non-motile, and rod-shaped strains, labelled as ProA8^T^ and ProA11^T^, were isolated from digestive tract of red snow crab (*Chionoecetes japonicus*). Strain ProA8^T^ grow at temperature range of 15–35°C, while strain ProA11^T^ grow at temperature range of 15–40°C. Phylogenetic and 16S rRNA gene sequence analysis revealed that strains ProA8^T^ and ProA11^T^ belonged to the genera *Microbacterium* and *Agrococcus*, respectively. Strain ProA8^T^ was closely affiliated with *Microbacterium yannicii* JCM 18959^T^ (98.8%) and strain ProA11^T^ was most closely related to *Agrococcus baldri* IAM 15147^T^ (98.9%). The genome sizes of ProA8^T^ and ProA11^T^ were 4,373,776 bp and 2,665,899 bp, with DNA G+C contents of 70.5% and 70.1%, respectively. The genomic relatedness values of ProA8^T^ and ProA11^T^ with their respective reference strains were <32.0% (for digital DNA–DNA hybridization) and <87.0% (for average nucleotide identity). Biosynthetic gene analysis showed the presence of genes for resorcinol production in ProA8^T^ and ectoine biosynthesis in ProA11^T^, indicating ecological and biotechnological significance. Virulence analyses determined that strains ProA8^T^ and ProA11^T^ were non-pathogenic to humans, highlighting their safe application in biotechnological field. The major cellular fatty acids reported in ProA8^T^ and ProA11^T^ were anteiso-C_15:0_, iso-C_16:0_, and anteiso-C_17:0_. Overall, the polyphasic taxonomic data suggest that both strains ProA8^T^ and ProA11^T^ represent two novel species within the genera *Microbacterium* and *Agrococcus*, respectively. Accordingly, we propose the names *Microbacterium chionoecetis* sp. nov., with the type strain ProA8^T^ (= KCTC 49861^T^ = JCM 37392^T^) and *Agrococcus chionoecetis* sp. nov., with the type strain ProA11^T^ (= KCTC 49958^T^ = JCM 37393^T^).

## Introduction

The marine gut microbial community provides valuable insights into both ecological processes and biotechnological advancements [1, 2]. These microbes play crucial roles in maintaining various biogeochemical cycles, supporting host health, sustaining marine food web dynamics, and enhancing resilience to environmental stressors [3]. Biotechnologically, marine gut microbiota offer valuable resources for industrial applications, including enzymes production, probiotics development, antibiotics discovery, and microbial solutions for bioremediation of hazardous pollutants [4-6]. The diverse range of marine microbes represents an untapped reservoir of genetic and functional potential, enabling advancements in synthetic biology, eco-friendly technologies, and the sustainable management of marine ecosystems [7, 8]. Therefore, exploring marine gut microbiota is essential for harnessing their potential as valuable industrial bioresources and understanding their ecological significance.

In this study, bacterial strains ProA8^T^ and ProA11^T^ in the genera *Microbacterium* and *Agrococcus*, respectively, were isolated from digestive tract of marine red snow crab (*Chionoecetes japonicus*). The genus *Microbacterium* was first proposed by Orla-Jensen et al. in 1919, with *Microbacterium lacticum* as the type species [9]. Later, its description was emended subsequently [10-12]. The genus *Microbacterium* is a member of the family *Microbacteriaceae* within the phylum *Actinomycetota*. Currently, there are 157 species with the correct names and valid publications accommodated in the genus *Microbacterium* (accession date: February 05, 2025; https://lpsn.dsmz.de/genus/microbacterium). Similarly, the genus *Agrococcus*, also a member of the family *Microbacteriaceae* within the phylum *Actinomycetota*, was delineated by Groth *et al*. with *Agrococcus jenensis* as the type species [13]. At present, a total of 11 species with the correct names and valid publications are included in the genus *Agrococcus* (accession date: February 05, 2025; https://lpsn.dsmz.de/genus/agrococcus). Members of the genera *Microbacterium* and *Agrococcus* have been isolated from a wide range of environments, such as soil [14, 15], sediment [16], air [17], water [18], compost [13], lung aspirates [19], faeces [20], gut [21], cow dung [22], sludge [23], cheeses [24], and seaweed [25]. Members of genera *Microbacterium* and *Agrococcus* have been also widely isolated from plant materials which are commonly referred as endophytic actinobacteria [10, 26, 27]. This study reports on the taxonomic assignment of strains ProA8^T^ and ProA11^T^ in the genera *Microbacterium* and *Agrococcus*.

## Materials and Methods

### Isolation of Strains

Strains ProA8^T^ and ProA11^T^ were isolated from intestinal samples collected from digestive tract of red snow crab (*Chionoecetes japonicus*) in Republic of Korea (36°51'35.7"N 128°26'53.0"E). To avoid cross-contamination, all equipments and utensils were sterilized using autoclaving or chemical treatments. Gloves were worn throughout the sample collection process. After collection, the samples were immediately transported to the laboratory under controlled conditions, stored at 4°C, and processed within a short time frame. The intestinal samples were serially diluted using sterile phosphate-buffered saline (pH 7.4). The samples were serially diluted and streaked onto marine agar 2216 (MA; Difco, USA) and incubated at 28°C for 3days. After incubation, isolated colonies were picked and streaked repeatedly on MA agar. Two pure colonies, designated as ProA8^T^ and ProA11^T^ were obtained and then preserved in a glycerol stock at temperature of -80°C.

### 16S rRNA and Phylogenetic Analysis

Genomic DNA from strains ProA8^T^ and ProA11^T^ was extracted using HiGene^TM^ Genomic DNA Prep Kit (BIOFACT, Republic of Korea). The 16S rRNA gene was amplified using forward (27F) and reverse primers (1492R) [28]. The amplified 16S rRNA gene was then sequenced as described previously [29]. The closest taxonomic relatives were identified by analysing the 16S rRNA nucleotide sequences in the EzBioCloud server [30]. Phylogenetic trees were constructed with MEGA X software [31] using maximum–likelihood (ML) [32], neighbour–joining (NJ) [33], and maximum-parsimony (MP) algorithms [34]. For the neighbour-joining tree, Kimura's two-parameter model was applied as a substitution model. A test of phylogeny was performed by a bootstrap method using 1,000 replications.

### Genome Analysis

The genomes of strains ProA8^T^ and ProA11^T^ were sequenced using PacBio RSII sequencing technology and raw sequences were assembled with HGAP v. 3.0 [35]. The quality of genome data was assured by analysing with ContEst16S algorithm [36], Blast-N tool [37], and CheckM v12.2 [38]. The genome was annotated using the Rapid Annotation Subsystem Technology (RAST) server [39] and the Prokaryotic Genome Annotation Pipeline (PGAP) [40]. Moreover, functional annotation was conducted by using the eggNOG 4.5 database [41]. The comparisons of orthologous gene clusters between strains ProA8^T^ and ProA11^T^ and closely related strains were executed using OrthoVenn3 v3.2.1 [42]. Pathogenicity potential and virulence factors of ProA8^T^ and ProA11^T^ were investigated with VirulenceFinder 2.0 [43, 44] and PathogenFinder 1.1 [45]. Antimicrobial resistance genes were explored by the Comprehensive Antibiotic Resistance Database (CARD) tool [46]. Gene clusters for secondary metabolites were explored with antiSMASH v5.0.1 [47]. DNA G+C content was estimated from the genome sequence data. The overall genome relatedness index (OGRI) between ProA8^T^ and ProA11^T^ and reference strains was computed by the Genome-to-Genome Distance Calculator [48] and an average nucleotide identity (ANI) tool [49]. The phylogenomic tree was constructed on the Type Strain Genome Server [50] using FastME 2.1.6.1 tools [51].

### Morphological, Biochemical, and Chemotaxonomic Analysis

The colony morphologies of strains ProA8^T^ and ProA11^T^ were determined by observing the colonies grown on MA agar at 28°C for 5 days. Gram staining was performed using the Color Gram 2 Kit (bioMérieux, USA). Catalase and oxidase activities, motility, and anaerobic growth ability of both strains were analysed as described [29]. Growth pH range and temperature were evaluated as mentioned previously [52]. Tests of biochemical, and carbon assimilation characteristics were conducted using commercial kits (API ZYM, API 20NE, and API 50 CH, bioMérieux). Cellular fatty acids were analyzed after cultivating strains ProA8^T^ and ProA11^T^ and reference species on R2A agar at 25°C for 3 days. Fatty acid extraction, analysis, and identification were performed following MIDI protocol [53]. Polar lipids and quinones were analyzed from freeze-dried cells [54, 55]. Polar lipids were visualized by spraying with different reagents [56].

## Results and Discussion

**Fig. 1:**
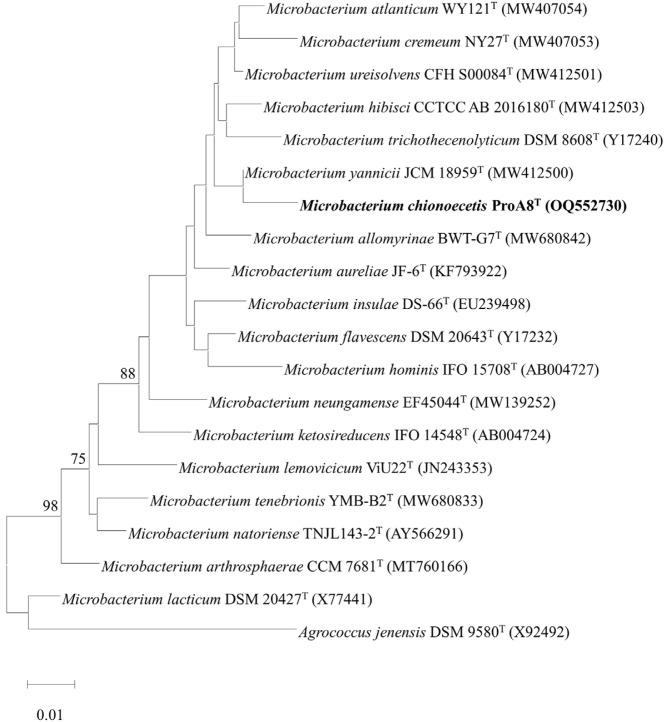
Maximum-likelihood tree generated based on the 16S rRNA gene sequences of strains ProA8^T^ and related reference taxa. The numbers at the branching nodes indicate the percentage of 1,000 bootstrap replications (only values >70% are shown). GenBank accession numbers for 16S rRNA gene sequences are provided in parentheses. The scale bar corresponds to 0.01 substitutions per nucleotide position. *Agrococcus jenensis* DSM 9580^T^ was used as an out-group.

**Fig. 2:**
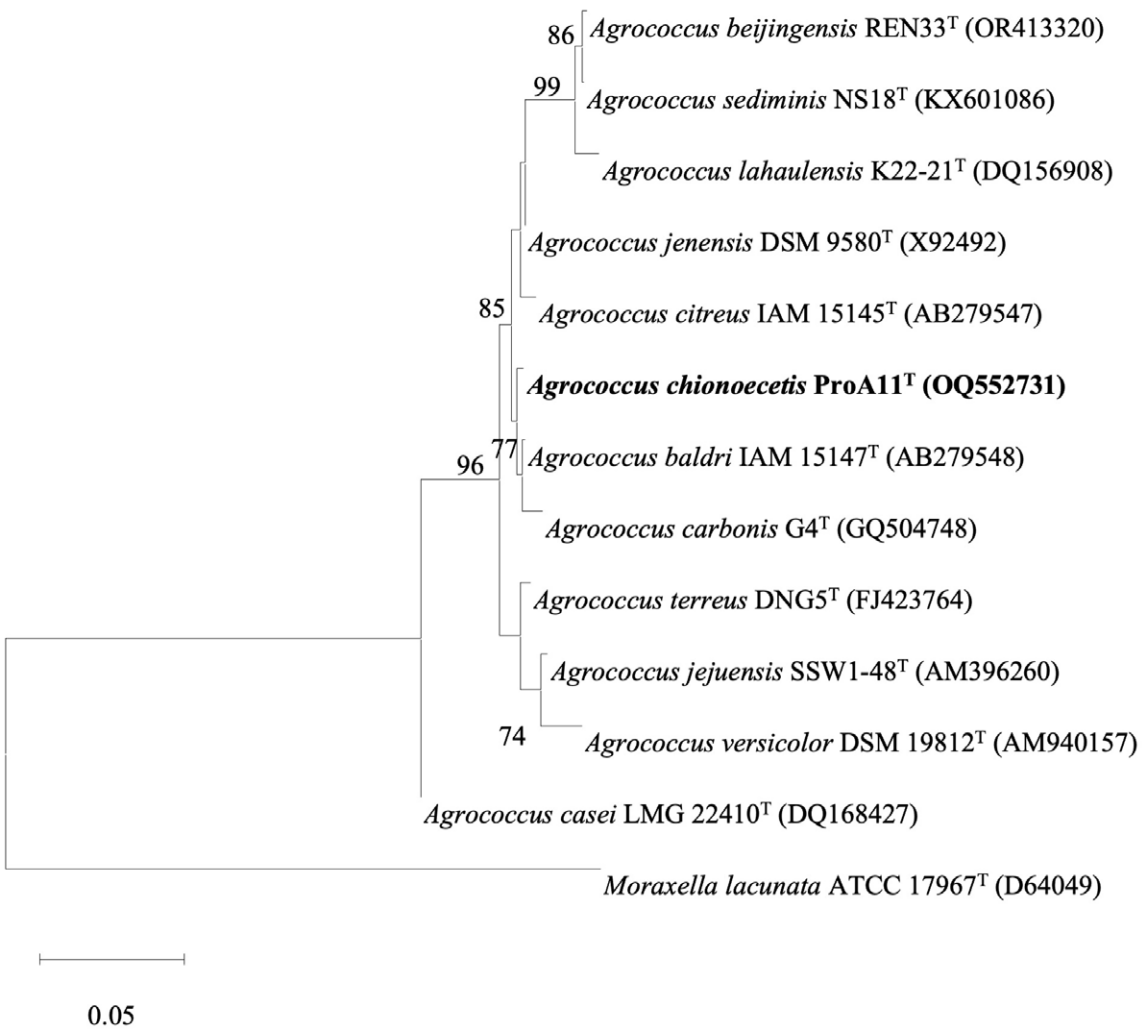
Maximum-likelihood tree generated based on the 16S rRNA gene sequences of strains ProA11^T^ and related reference taxa. The numbers at the branching nodes indicate the percentage of 1,000 bootstrap replications (only values >70% are shown). GenBank accession numbers for 16S rRNA gene sequences are provided in parentheses. The scale bar corresponds to 0.05 substitutions per nucleotide position. *Moraxella lacunata* ATCC 17967^T^ was used as an out-group.

The lengths of 16S rRNA gene sequences of strains ProA8^T^ and ProA11^T^ were 1,375 bp and 1,354 bp, respectively. The 16S rRNA gene sequence comparison with the reference database revealed that strains ProA8^T^ and ProA11^T^ belonged to the genera *Microbacterium* and *Agrococcus*, respectively. Strain ProA8^T^ reported highest 16S rRNA gene sequence similarities with *Microbacterium yannicii* JCM 18959^T^ (98.8%), *Microbacterium arthrosphaerae* CC-VM-YT (98.6%), and *Microbacterium hominis* IFO 15708^T^ (98.3%), while strain ProA11^T^ exhibited highest affiliation with *Agrococcus baldri* IAM 15147^T^ (98.9%), *Agrococcus jenensis* DSM 9580^T^ (98.7%), and *Agrococcus carbonis* G4^T^ (98.7%). The 16S rRNA gene nucleotide sequence identities of both strains with all reference taxa except for *M. yannicii* JCM 18959^T^ (98.8%), and *Agrococcus baldri* IAM 15147^T^ (98.9%) were lower than the specified cut-off values of < 98.7% (Table 1), which are generally used for species differentiation [57, 58]. The species discrimination with the exceptional species (> 98.7%) was confirmed by genomic analysis. This suggests that both strains ProA8^T^ and ProA11^T^ represent novel species within the genera *Microbacterium* and *Agrococcus*, respectively. All phylogenetic trees (ML, NJ, and MP) formed a clade among strain ProA8^T^ and *M. yannicii* JCM 18959^T^ and *M. hominis* IFO 15708^T^ (Figs. 1, S1, and S2). Similarly, both ML and NJ trees constructed a clade between strain ProA11^T^ and reference type strains *A. baldri* IAM 15147^T^ and *A. carbonis* G4^T^ (Figs. 2 and S3). However, MP tree generated a distinct lineage for strain ProA11^T^ within the species of the genus *Agrococcus* (Fig. S4). Strain ProA11^T^ may reflect a unique evolutionary trajectory, making it phylogenetically distinct in MP tree. However, the ML and NJ trees may suggest that strain ProA11^T^ still shares substantial genetic similarities with other species of *Agrococcus*.

CheckM, BLASTn, and ContEst16S analyses of the genome sequences of strains ProA8^T^ and ProA11^T^ confirmed that the resulting genome sequences were valid with completeness of 99.1% and contamination of 0.5%. The genomes of strains ProA8^T^ and ProA11^T^ were 4,373,776 bp and 2,665,899 bp with DNA G+C contents of 70.5% and 71.0%, respectively. The genome of both strains was assembled into 1 scaffold with genome coverages of 153.3x for ProA8^T^ and 283.6x for ProA11^T^ (Table S1). The genome annotation of ProA8^T^ and ProA11^T^ conducted by RAST determined a total of 277 and 236 subsystem features, respectively (Figs. S5 and S6). The dDDH and ANI values of ProA8^T^ and ProA11^T^ with the nearest phylogenetic taxa ranged between 19.6–32.0%and 73.3–86.7%, respectively (Table 1). These OGRI values were lower than the threshold values of dDDH (70.0%) and ANI (95.0%), indicating that strains ProA8^T^ and ProA11^T^ are distinct from the other members of genera *Microbacterium* and *Agrococcus*, respectively [59, 60]. The phylogenomic tree exhibited that strain ProA8^T^ generated a clade with *M. yannicii* DSM 23203^T^ (Fig. S7), while that of strain ProA11^T^ clustered with *A. baldri* NBRC 103055^T^ and *A. carbonis* DSM 22965^T^ (Fig. S8).

**Fig. 3:**
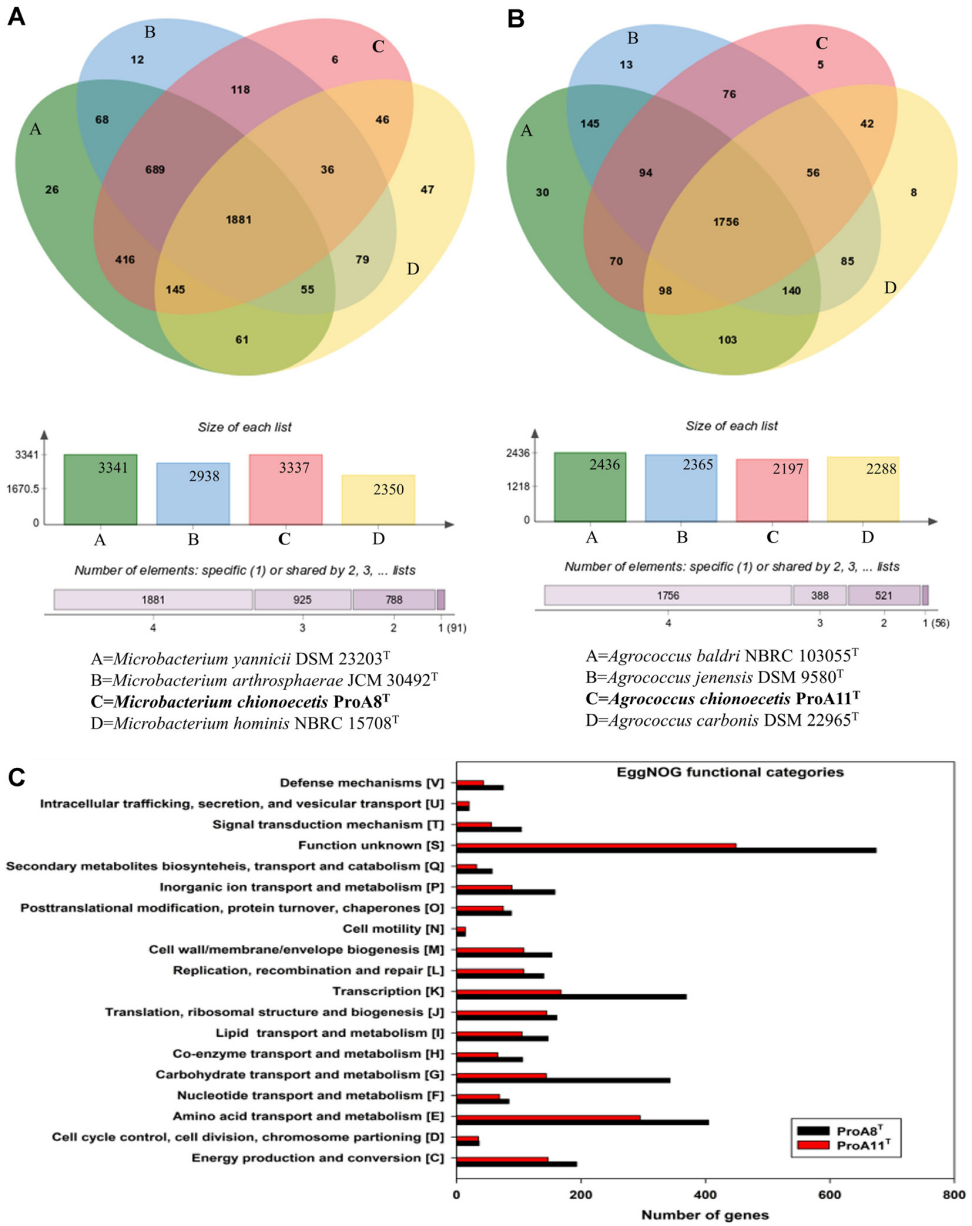
Venn diagrams of orthologous genes and distribution of gene clusters into EggNOG functional categories of strains ProA8^T^ and ProA11^T^. (**A**) Venn diagram of orthologous genes of strain ProA8^T^. (**B**) Venn diagram of orthologous genes of strains ProA11^T^ and (**C**) EggNog functional categories.

In both strains ProA8^T^ and ProA11^T^, biosynthetic gene cluster analysis showed the presence of several genes encoding for terpene, betalactone, and type III polyketide synthase. Genes responsible for resorcinol production were identified in the genome of ProA8^T^, whereas genes clusters encoding ectoine were observed in ProA11^T^ (Table S2). Resorcinol helps bacteria compete in complex microbial ecosystems by inhibiting competing species. Resorcinol also act as precursor for antiseptics and anti-inflammatory drugs [61]. Ectoine helps to protect against osmotic stress, heat, and desiccation. Ectoine can also be used as a stabilizer, moisturizer, and anti-inflammatory agent [62, 63]. The Venn diagram (Fig. 3A) revealed that a total of 1,881 orthologous genes were shared between strain ProA8^T^, *M. yannicii* DSM 23203^T^, *M. arthrosphaerae* JCM 30492^T^, and *M. hominis* NBRC 15708^T^; of these, 416 genes were only shared with *M. yannicii* DSM 23203^T^. Similarly, a total of 1,756 orthologous genes were shared between strain ProA11^T^, *A. baldri* NBRC 103055^T^, *A. jenensis* DSM 9580^T^, and *A. carbonis* DSM 22965^T^; of these, 70 genes were only shared with *A. baldri* NBRC 103055^T^ (Fig. 3B). A total of 3,327 and 2,168 genes of strains ProA8^T^ and ProA11^T^ were grouped into 19 functional categories of the orthologous gene clusters of eggNOG pipeline, respectively. The highest number of genes were grouped into the unknown functional category [S], followed by amino acid transport and metabolism [E] (Fig. 3C). Both Pathogen Finder and Virulence Finder tools determined that strains ProA8^T^ and ProA11^T^ were likely to be non-pathogenic to humans. The CARD tool did not find any gene encoding for antibiotic resistance.

Annotation results presented in Table S3 highlighted that the genomes of both strains ProA8^T^ and ProA11^T^ contained several genes that encode various functional proteins. Genes responsible for glycoside hydrolase proteins, which mediate the breakdown of complex carbohydrates, were found in both strains [64]. For low-temperature adaptation and oxidative response, various genes encoding cold-shock proteins, catalase/peroxidase HPI, catalase family protein, and glutathione peroxidase, were detected in the genomes of both strains. Pyrroline-5-carboxylate reductase was reported from both strains ProA8^T^ and ProA11^T^. Pyrroline-5-carboxylate reductase is an enzyme involved in proline metabolism and is related to defense mechanisms and abiotic stress-alleviating phenomena in plants [65]. For osmoregulation, ProA8^T^ contained aquaporin Z and ProA11^T^ harboured MIP/aquaporin family protein. Furthermore, both strains contained several genes associated to plant growth promotion. The annotation data exhibited proteins involved in ammonia assimilation, auxin biosynthesis, phosphate solubilization, and siderophore production. Similarly, genes encoding biotin and folate biosynthesis proteins were also noted from genomes of both strains. Overall, the genome annotation of both strains ProA8^T^ and ProA11^T^ displayed various genes which are crucial for ecophysiological functions, cell adaptation, biotechnological application, and plant growth promotion.

Strains ProA8^T^ and ProA11^T^ were non-motile, Gram-stain-positive, and rod-shaped. Strain ProA8^T^ grow at temperature range of 15–35°C and pH range of 5.0–8.5; while strain ProA11^T^ grow at temperature range of 15–40°C and pH range of 5.0–9.5. Both strains exhibited oxidase and urease negative. Catalase activity and gelatin hydrolysis were positive for strain ProA11^T^, but negative for strain ProA8^T^. Esculin was hydrolysed by both strains. Esterase lipase (C4) was positive for ProA8^T^, but was negative for other nearest reference strains. *β*-glucuronidase was negative for ProA8^T^, while closest reference taxa revealed positive. Strain ProA11^T^ exhibited positive for cystine arylamidase, but was negative for other reference species. Other major differentiating properties of ProA8^T^ and ProA11^T^ are provided in the species description and presented in Table 2 along with the closest reference strains. All the enzymatic and carbon assimilative traits obtained from API ZYM, API 20NE, and API 50 CH are illustrated in Table S4.

Strain ProA8^T^ detected MK-12 (76.9.8%), MK-13 (16.8%), and MK-11 (6.3%) as the major menaquinones, which is similar with other members of the genus *Microbacterium* [10, 21]. Similarly, strain ProA11^T^ showed MK-10 (45.6%), MK-9 (32.3%), and MK-11 (22.1%) as the predominant menaquinones, which align with other taxa of the genus *Agrococcus* [14, 66]. The major cellular fatty acids found in both strains were anteiso-C_15:0_ (38.3% for ProA8^T^ and 50.3% for ProA11^T^), anteiso-C_17:0_ (31.6% for ProA8^T^ and 30.3% for ProA11^T^), and iso-C_16:0_ (11.4% for ProA8^T^ and 6.7% for ProA11^T^). While the main fatty acids of ProA8^T^ and ProA11^T^ were similar, minor fatty acids were proportionally and/or compositionally different from those of the phylogenetically affiliated species (Table 3). The minor fatty acid iso-C_18:0_ was detected in both strains ProA8^T^ and ProA11^T^ but was absent in other closely related reference species. Furthermore, C_17:0_, anteiso-C_15:1_ A, and iso-C_17:0_ 3OH were found exclusively in strain ProA8^T^ and not observed in its closest relatives. Phosphatidylglycerol (PG) and diphosphatidylglycerol (DPG) were major polar lipids in both strains. Additionally, one unidentified glycolipid (GL1) was also identified in strain ProA8^T^ as major polar lipid and two unidentified glycolipids (GL1-GL2) were detected in strain ProA11^T^ as major polar lipids (Fig. S9). The polar lipids profiles of strains ProA8^T^ and ProA11^T^ are similar to other members of the genera *Microbacterium* and *Agrococcus* [13, 14, 27].

### Taxonomic Conclusion

In conclusion, the polyphasic taxonomic results presented here confirmed that strains ProA8^T^ and ProA11^T^ are a new species of the genera *Microbacterium* and *Agrococcus*, respectively, for which the names *Microbacterium chionoecetis* sp. nov. and *Agrococcus chionoecetis* sp. nov. are proposed, respectively.

### Description of *Microbacterium chionoecetis* sp. nov.

*Microbacterium chionoecetis* sp. nov. (chi.on.oe.cétis. N.L. gen. n. *chionoecetis*, of *Chionoecetes*, a crab genus from which strain was isolated).

Cells are aerobic, Gram-stain-positive, non-motile, and rod-shaped. Colonies are yellow-coloured, smooth, convex, and punctiform with the diameter of 0.6–0.8 mm. Cells grow at temperature of 15–35°C (optimum 28°C–30°C) and at pH of 5.0–8.5 (optimum 6.5). Negative for nitrate reduction, oxidase test, and catalase activity. Hydrolyse esculin but cannot hydrolyse urea and gelatin. Positive for esterase (C4), alkaline phosphatase, esterase lipase (C8), leucine arylamidase, *α*-chymotrypsin, valine arylamidase, acid phosphatase, *α*-galactosidase, *β*-galactosidase, naphthol-AS-BI-phosphohydrolase, *α*-glucosidase, *β*-glucosidase, and *N*–Acetyl -*β*-glucosaminidase. Weakly positive for cystine arylamidase, trypsin, and *α*-mannosidase. Assimilates *β*-galactosidase (PNPG), glycerol, erythritol, L-arabinose, D-arabinose, D-ribose, methyl *β*-D-xylopyranoside, D-adonitol, D-glucose, D-galactose, D-mannose, D-fructose, L-rhamnose, dulcitol, inositol, D-sorbitol, methyl *α*-D-mannopyranoside, D-mannitol, methyl *α*-D-glucopyranoside, amygdalin, arbutin, *N*-acetylglucosamine, esculin, ferric citrate, D-cellobiose, D-maltose, salicin, D-melibiose, D-trehalose, D-melezitose, D-saccharose, amidon, D-raffinose, and glycogen. Weakly assimilates D-turanose, D-lyxose, D-tagatose, D-fucose, L-fucose, D-arabitol, and L-arabitol. The main menaquinones are MK-12, MK-13, and MK-11, major polar lipids are DPG, PG, and GL1, and major cellular fatty acids are anteiso-C_15:0_, iso-C_16:0_, and anteiso-C_17:0_. DNA G+C content is 70.5%.

The type strain, ProA8^T^ (= KCTC 49861^T^ = JCM 37392^T^), was isolated from soil in Republic of Korea (36°51'35.7"N 128°26'53.0"E).

The GenBank/EMBL/DDBJ accession numbers for the 16S rRNA gene and genome sequences of strain ProA8^T^ are OQ552730 and CP157000, respectively.

### Description of *Agrococcus chionoecetis* sp. nov.

*Agrococcus chionoecetis* sp. nov. (chi.on.oe.cétis. N.L. gen. n. *chionoecetis*, of *Chionoecetes*, a crab genus from which strain was isolated).

Cells are aerobic, Gram-stain-positive, non-motile, and rod-shaped. Colonies are yellow-coloured, smooth, convex, and circular with the diameter of 1.0–1.2 mm. Cells grow at temperature of 15–40°C (optimum 28°C–30°C) and at pH of 5.0–9.5 (optimum 6.5). Negative for nitrate reduction and oxidase tests. Positive for catalase test. Hydrolyse esculin and gelatin but cannot hydrolyse urea. Positive for leucine arylamidase, naphtol-AS-BI-phosphohydrolase, and *α*-glucosidase. Weakly positive for esterase (C4), valine arylamidase, cystine arylamidase, esterase lipase (C8), and acid phosphatase. Assimilates *β*-galactosidase (PNPG), D-mannitol, D-maltose, gluconate, and esculin. Weakly assimilates D-arabinose, D-glucose, D-mannose, L-rhamnose, D-fructose, D-mannitol, salicin, D-cellobiose, D-melibiose, and gentiobiose. The main menaquinones are MK-10, MK-9, and MK-11, major polar lipids are DPG, PG, GL1 and GL2, and major cellular fatty acids are anteiso-C_15:0_, iso-C_16:0_, and anteiso-C_17:0_. DNA G+C content is 71.0%.

The type strain, ProA11^T^ (=KCTC 49958^T^ = JCM 37393^T^), was isolated from soil in Republic of Korea (36°51'35.7"N 128°26'53.0"E).

The GenBank/EMBL/DDBJ accession numbers for the 16S rRNA nucleotide and genome sequences of strain ProA11^T^ are OQ552731 and CP156989, respectively.

## Supplemental Materials

Supplementary data for this paper are available on-line only at http://jmb.or.kr.



## Figures and Tables

**Table 1:** 16S rRNA similarity, digital DNA-DNA hybridization (dDDH), and average nucleotide identity (ANI) values of strains ProA8^T^ and ProA11^T^ with their respective reference strains.

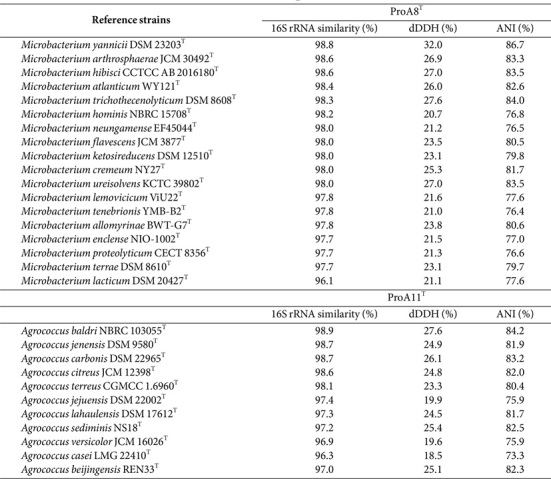

**Table 2:** Differentiating properties of strains ProA8^T^ and ProA11^T^ and related reference members.

Characteristic	1	2	3	4	5	6	7	8
Growth temperature (°C)	15–35	4–37	15–40	10–42	15–40	4–35	15–35	15–37
pH range	5.0–8.5	5.5–9.0	5.5–10.0	5.5–11.0	5.0–9.5	5.0–10.0	5.5–10.0	5.0–10.5
Catalase	-	+	+	+	+	+	+	+
oxidase	-	-	+	-	-	-	-	-
Nitrate reduction	-	-	-	-	+	-	-	-
Hydrolysis of								
Esculin	+	w	+	+	+	-	+	+
Gelatin	-	+	-	-	+	-	-	-
Enzyme activity								
Esterase Lipase (C4)	+	-	-	-	w	-	-	+
Esterase Lipase (C8)	+	+	-	-	w	-	+	+
Leucine arylamidase	+	+	-	+	+	+	+	-
Valine arylamidase	+	+	-	+	w	+	w	+
Cystine arylamidase	w	+	-	-	+	-	-	+
*α*-Chymotrypsin	+	+	-	+	-	+	+	-
*α*-Galactosidase	+	+	+	-	-	-	+	-
*β*-Galactosidase	+	+	+	+	-	-	+	-
*β*-Glucuronidase	-	+	+	-	-	-	-	-
*α*-Glucosidase	+	+	-	+	+	+	-	+
*β*-Glucosidase	+	+	+	+	-	-	-	-
*N*–Acetyl -*β*-glucosaminidase	+	+	+	+	-	-	+	+
DNA G + C content (%)	70.5	70.0	71.0	71.0	71.0	72.0	72.5	73.0

Strains: 1, ProA8^T^; 2, *M. yannicii* KACC 17732^T^; 3, *M. arthrosphaerae* KACC 16680^T^; 4, *M. hominis* KACC 14459^T^; 5, ProA11^T^; 6, *A. baldri* KACC 11969^T^ ; 7, *A. jenensis* KACC 20580^T^; 8, *A. carbonis* DSM 22965^T^. All data were obtained in this study. The G+C content data were computed from genome sequence of respective strains. +, positive; w, weakly positive; -, negative.

**Table 3:** Cellular fatty acid profiles (% of totals) of ProA8^T^ and ProA11^T^, and related reference members.

Fatty acid	1	2	3	4	5	6	7	8
Saturated								
C_16:0_	tr	tr	3.4	3.8	TR	3.8	2.4	1.1
C_17:0_	1.0	–	–	1.2	tr	–	–	–
C_18:0_	tr	1.0	1.1	tr	–	–	–	–
Unsaturated								
C_18:1_ ω5c	–	–	–	–	2.5	1.5	2.1	1.7
Branched saturated								
iso-C_14:0_	1.2	tr	1.5	tr	tr	–	1.0	2.1
iso-C_15:0_	2.1	4.3	5.1	3.5	3.2	4.8	11.4	6.1
iso-C_16:0_	**11.4**	**11.2**	**13.2**	**14.1**	**6.7**	**8.6**	**9.8**	**7.4**
iso-C_17:0_	5.3	4.6	4.2	2.0	1.0	2.9	3.1	1.8
iso-C_18:0_	3.1	2.0	–	–	tr	–	–	–
anteiso-C_15:0_	**38.3**	**41.2**	**40.8**	**42.5**	**50.3**	**46.8**	**52.5**	**45.7**
anteiso-C_15:1_ A	2.0	–	–	–	–	–	–	–
anteiso-C_17:0_	**31.6**	**32.7**	**30.5**	**29.7**	**30.3**	**27.1**	**14.6**	**28.4**
iso-C_17:0_ 3OH	1.1	–	–	–	–	–	–	–
Summed features*								
5	–	–	–	–	1.8	1.0	2.4	1.9

Strains: 1, ProA8^T^; 2, *M. yannicii* KACC 17732^T^; 3, *M. arthrosphaerae* KACC 16680^T^; 4, *M. hominis* KACC 14459^T^; 5, ProA11^T^; 6, *A. baldri* KACC 11969^T^ ; 7, *A. jenensis* KACC 20580^T^; 8, *A. carbonis* DSM 22965^T^. All data were obtained in this study. TR, trace amount (<1.0%); –, not detected.

*Summed features represent groups of two or three fatty acids that could not be separated using the MIDI system. Summed feature 5 comprised C_18:2_*w6,9c* and/or anteiso-C_18:0_. Unknown fatty acids are designated by their ECL, relative to the chain lengths of known straight-chain saturated fatty acids.
