# Supplementary material for: Microbacterium chionoecetis sp. nov. and Agrococcus chionoecetis sp. nov.: Novel Gut Bacteria from Red Snow Crab
Source: J Microbiol Biotechnol. 2025 Apr 24;35:e2412044. doi: 10.4014/jmb.2412.12044 (PMC12089952; doi:10.4014/jmb.2412.12044)
Supplement: Supplementary file 1 [file jmb-35-e2412044-supple.pdf]

J. Microbiol. Biotechnol. 2025. 35: e2412044
https://doi.org/10.4014/jmb.2412.12044

Supplementary Tables and Figures

Microbacterium chionoecetis sp. nov. and *Agrococcus chionoecetis* sp. nov.: Novel gut bacteria from red snow crab

Dhiraj Kumar Chaudhary [1,2]†, Sang-Eon Kim [1,2]†, Hye-Jin Park[3], and Kyoung-Ho Kim [1,2]*

[1] Department of Microbiology, Pukyong National University, Busan, 48513, Republic of Korea [2] Division of Marine and Fisheries Life Sciences, Pukyong National University, Busan, 48513, Republic of Korea [3] Korea Institute of Ocean Science and Technology, Busan, Republic of Korea

Short title: *Microbacterium chionoecetis* sp. nov. and *Agrococcus chionoecetis* sp. nov.

†The authors equally contributed to this work

* Corresponding author e-mail: kimkh@pknu.ac.kr

Table S1. The **draft genome features of strains ProA8^{T} and ProA11^{T}.**

**Genome features**	**ProA8^{T}**	**ProA11^{T}**
Genome size (bp)	4,373,776	2,665,899
G + C content (%)	70.5	71.0
No. of contigs	1	1
N50	4,373,776	2,665,899
L50	1	1
No. of subsystem	277	236
No. of proteins	3,845	2,483
Total genes	3,913	2,502
CDSs (total)	3,857	2,502
Protein-coding genes	3,845	2,483
Genes (RNA)	56	51
rRNAs (5S, 16S, 23S)	2, 2, 2	1, 1, 1
Complete rRNAs (5S)	2, 2, 2	1, 1, 1
tRNAs	47	45
ncRNAs	3	3
Pseudo Genes (total)	12	19
Genome coverage	135.3x	283.6x

Table S2. The distribution of biosynthetic gene clusters (BGCs) in the draft genome of the strains ProA8^T and ProA11^T.

ProA8^T					
**Genomic regions**	**Type**	**From**	**To**	**Most similar known cluster**	**Similarity (%)**
Region 1	RRE-containing	363,322	383,582		
Region 2	Betalactone	1,809,880	1,837,843	Microansamycin	7
Region 3	Terpene	2599189	2,620,070	Carotenoid	21
Region 4	Resorcinol	3,576,509	3,617,651	Azicemicin B	4
Region 5	T3PKS	4,123,081	4,164,238	Formicamycins A-M	4
**ProA11^T**					
**Genomic regions**	**Type**	**From**	**To**	**Most similar known cluster**	**Similarity (%)**
Region 1	Terpene	82,322	103,230	Carotenoid	35
Region 2	T3PKS	148,530	189,714	Thermochelin	15
Region 3	Betalactone	1,648,265	1,674,044	Microansamycin	7
Region 4	Ectoine	2,133,223	2,143,606	Ectoine	75
Region 5	RiPP-like	2,267,858	2,278,136		

RRE: RiPP recognition element; RiPP: Ribosomally synthesised and post-translationally modified peptide product; T3PKS: Type III PKS; PKS: Polyketide synthase.

Table S3. Presence of various genes that encode the different proteins associated with adaptation, eco-physiological and biotechnological roles, plant growth promotion, and antimicrobial resistance in the draft genome of strains ProA8^T and ProA11^T.

**ProA8^T**			
**Functions**	**Gene annotation**	**Protein accession**	**Locus tag**
**Adaptation and eco-physiological roles**			
Breakdown of complex plant-derived carbohydrates	glycoside hydrolase	WP_347977967.1	ABG085_RS03015
	glycoside hydrolase family 3 N-terminal domain-containing protein	WP_347979254.1	ABG085_RS01240
	glycoside hydrolase family 78 protein	WP_347977757.1	ABG085_RS01910
	glycoside hydrolase family 3 N-terminal domain-containing protein	WP_347977949.1	ABG085_RS02910
Helps to adapt in cold environment	cold-shock protein	WP_137845695.1	ABG085_RS00145
	cold-shock protein	WP_026059428.1	ABG085_RS14225
	cold-shock protein	WP_137845695.1	ABG085_RS00145
	cold-shock protein	WP_026059428.1	ABG085_RS14225
Mediate oxidative response	catalase/peroxidase HPI	WP_171218527.1	ABG085_RS05815
	glutathione peroxidase	WP_347978237.1	ABG085_RS04530
Involved in proline metabolism	pyrroline-5-carboxylate reductase	WP_347976613.1	ABG085_RS15410
Involved in glycogen metabolism	glycogen synthase	WP_347978950.1	ABG085_RS08505
Osmoregulation	aquaporin Z	WP_347978475.1	ABG085_RS05820
**Plant growth promotion**			
Auxin biosynthesis	tryptophan synthase subunit alpha	WP_347979035.1	ABG085_RS08995
	tryptophan synthase subunit beta	WP_347979036.1	ABG085_RS09000
Ammonia assimilation	ammonium transporter	WP_347979090.1	ABG085_RS09325
Phosphate solubilization	pyruvate kinase	WP_347979032.1	ABG085_RS08975
	polyphosphate kinase 2 family protein	WP_347976565.1	ABG085_RS15195
Siderophore production	siderophore-interacting protein	WP_347977622.1	ABG085_RS01170

	iron-siderophore ABC transporter substrate-binding protein	WP_347978935.1	ABG085_RS08430
**Biotechnological and industrial application**			
Biotin biosynthesis	biotin transporter BioY	WP_347978936.1	ABG085_RS08435
	biotin--[acetyl-CoA-carboxylase] ligase	WP_347978435.1	ABG085_RS05580
	biotin/lipoyl-binding protein	WP_347978491.1	ABG085_RS05920
Folate Biosynthesis	folylpolyglutamate synthase/dihydrofolate synthase family protein	WP_347975596.1	ABG085_RS09985
	dihydrofolate reductase	WP_347975990.1	ABG085_RS12120
	5-formyltetrahydrofolate cyclo-ligase	WP_347976160.1	ABG085_RS13020
	folylpolyglutamate synthase/dihydrofolate synthase family protein	WP_347975596.1	ABG085_RS09985
**Antibiotic resistance**			
Vancomycin resistance	M15 family metallopeptidase	WP_347977619.1	ABG085_RS01155
**ProA11^{T}**			
**Functions**	**Gene annotation**	**Protein accession**	**Locus tag**
**Adaptation and eco-physiological roles**			
Breakdown of complex plant-derived carbohydrates	glycoside hydrolase family 13 protein	WP_347755968.1	ABG090_RS02110
	glycoside hydrolase family 15 protein	WP_347754483.1	ABG090_RS10775
	glycoside hydrolase family 99-like domain-containing protein	WP_347756279.1	ABG090_RS03015
Helps to adapt in cold environment	cold-shock protein	WP_072314408.1	ABG090_RS01825
Mediate oxidative response	catalase	WP_347755064.1	ABG090_RS12550
	glutathione peroxidase	WP_347754671.1	ABG090_RS11620
Involved in proline metabolism	pyrroline-5-carboxylate reductase	WP_347755911.1	ABG090_RS01925
Involved in glycogen metabolism	glycogen synthase	WP_347753619.1	ABG090_RS06640
Osmoregulation	MIP/aquaporin family protein	WP_347757545.1	ABG090_RS07095
**Plant growth promotion**			
Auxin biosynthesis	tryptophan synthase subunit alpha	WP_347757525.1	ABG090_RS06350
	tryptophan synthase subunit beta	WP_347757438.1	ABG090_RS06345
Ammonia assimilation	ammonium transporter	WP_347757513.1	ABG090_RS05930

Phosphate solubilization	pyruvate kinase	WP_347757446.1	ABG090_RS06370
Siderophore production	siderophore-interacting protein	WP_347757072.1	ABG090_RS05290
	siderophore-interacting protein	WP_347754000.1	ABG090_RS08200
**Biotechnological and industrial application**			
Biotin biosynthesis	biotin transporter BioY	WP_347753605.1	ABG090_RS06590
	biotin--[acetyl-CoA-carboxylase] ligase	WP_347756237.1	ABG090_RS02900
	biotin/lipoate A/B protein ligase family protein	WP_347754642.1	ABG090_RS11495
Folate Biosynthesis	folylpolyglutamate synthase/dihydrofolate synthase family protein	WP_347753836.1	ABG090_RS07565
	dihydrofolate reductase	WP_347754057.1	ABG090_RS08535
	5-formyltetrahydrofolate cyclo-ligase	WP_347754325.1	ABG090_RS09950
	folylpolyglutamate synthase/dihydrofolate synthase family protein	WP_347753836.1	ABG090_RS07565
**Antibiotic resistance**			
Vancomycin resistance	M15 family metallopeptidase	WP_347755854.1	ABG090_RS01755

Table S4. The enzymatic and assimilation data obtained from API ZYM, API 20NE and API 50 CH tests of strains ProA8^{T} and ProA11^{T}. Strains: 1, ProA8^{T}; 2, ProA11^{T}. +, positive; w, weakly positive; −, negative.

**API ZYM test**	**1**	**2**
Alkaline phosphatase	+	-
Esterase (C4)	+	w
Esterase Lipase (C8)	+	w
Lipase (C14)	-	-
Leucine arylamidase	+	+
Valine arylamidase	+	w
Cystine arylamidase	w	w
Trypsin	w	-
α-Chymotrypsin	+	-
Acid phosphatase	+	w
Naphtol-AS-BI-phosphohydrolase	+	+
α-Galactosidase	+	-
β-Galactosidase	+	-
β-Glucuronidase	-	-
α-Glucosidase	+	+
β-Glucosidase	+	-
N–Acetyl -*β*-glucosaminidase	+	-
α-Mannosidase	w	-
α-Fucosidase	-	-
**API 20NE test**		
Reduction of nitrates (NO_3^-) to nitrites (NO_2^-)	-	+
Reduction of nitrates (NO_3^-) to nitrogen(N_2)	-	-
Indole production	-	-
Glucose Acidification	-	-
Arginine dihydrolase	-	-
Urease	-	-
β-Glucosidase (esculin hydrolysis)	+	+
Protease (gelatin hydrolysis)	-	+
β-Galactosidase (PNPG)	+	+
D-Glucose	-	w
L-Arabinose	-	-
D-Mannose	-	-
D-Mannitol	-	+
N-Acetyl-D-glucosamine	-	-
D-Maltose	-	+
Gluconate	-	+
Caprate	-	-
Adipate	-	-
Malate	-	-

Citrate	-	-
Phenyl-acetate	-	-
**API 50 CH test**		
Glycerol	+	-
Erythritol	+	-
D-Arabinose	+	w
L-Arabinose	+	-
D-Ribose	+	-
D-Xylose	-	-
L-Xylose	-	-
D-Adonitol	+	-
Methyl *β*-D-xylopyranoside	+	-
D-Galactose	+	-
D-Glucose	+	w
D-Fructose	+	w
D-Mannose	+	w
L-Sorbose	-	-
L-Rhamnose	+	w
Dulcitol	+	-
Inositol	+	-
D-Mannitol	+	w
D-Sorbitol	+	-
Methyl *α*-D-Mannopyranoside	+	-
Methyl *α*-D-Glucopyranoside	+	-
N-Acetylglucosamine	+	-
Amygdalin	+	-
Arbutin	+	-
Esculin	+	+
Ferric citrate	+	-
Salicin	+	w
D-Cellobiose	+	w
D-Maltose	+	-
D-Lactose (bovine origin)	-	-
D-Melibiose	+	w
D-Saccharose (sucrose)	+	-
D-Trehalose	+	-
Inulin	-	-
D-Melezitose	+	-
D-Raffinose	+	-
Amidon (starch)	+	-
Glycogen	+	-
Xylitol	+	-
Gentiobiose	-	w
D-Turanose	w	-

D-Lyxose	w	-
D-Tagatose	w	-
D-Fucose	w	-
L-Fucose	w	-
D-Arabitol	w	-
L-Arabitol	w	-
Potassium gluconate	-	-
Potassium 2-ketogluconate	-	-
Potassium 5-ketogluconate	-	-

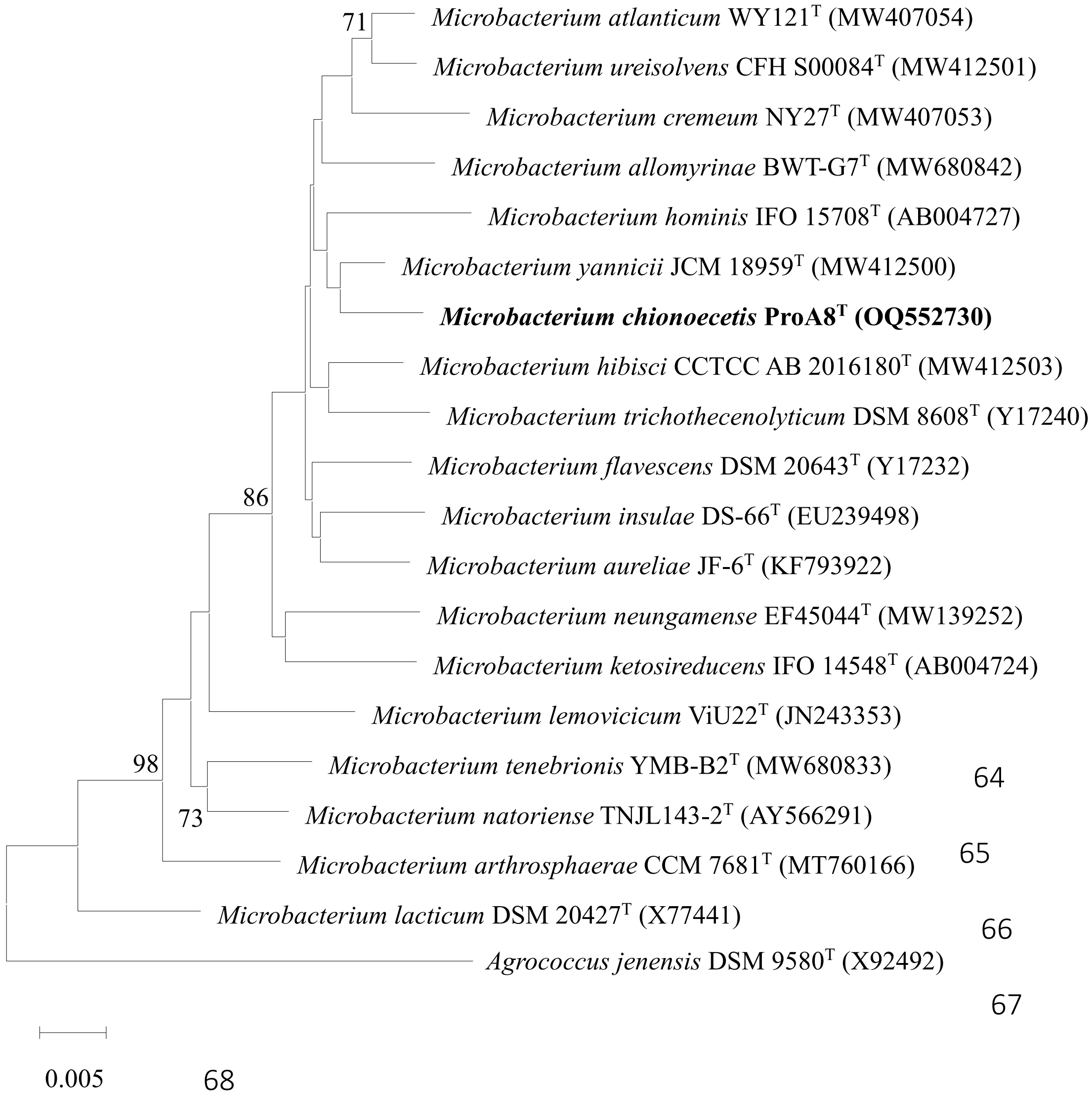

Fig. S1. Neighbor-joining tree generated based on the 16S rRNA gene sequences of strain ProA8^T and related reference taxa. The numbers at the branching nodes indicate the percentage of 1,000 bootstrap replications (only values >70% are shown). GenBank accession numbers for 16S rRNA gene sequences are provided in parentheses. The scale bar corresponds to 0.005 substitutions per nucleotide position**.** *Agrococcus jenensis* DSM 9580^T was used as an out-group.

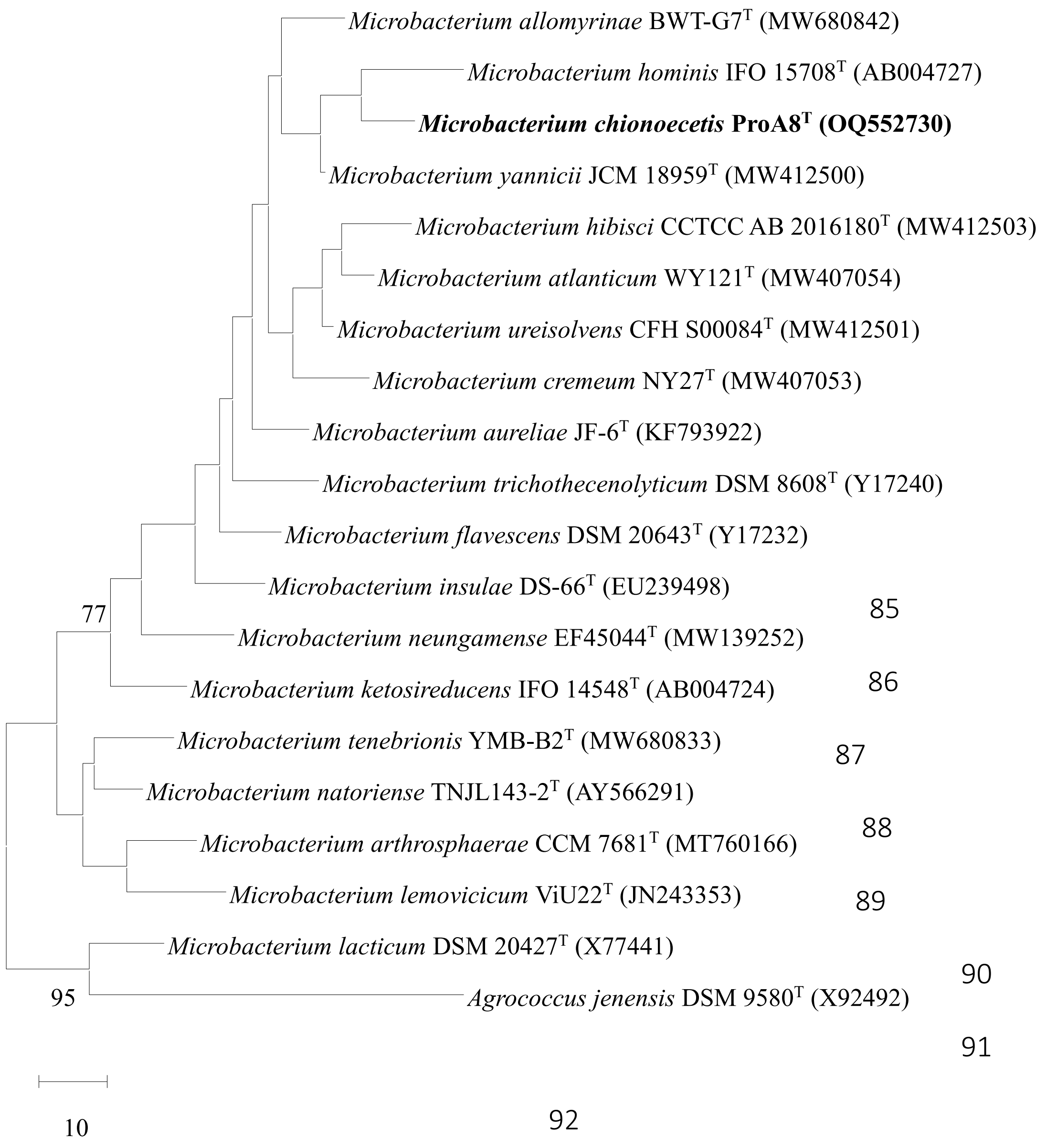

Fig. S2. Maximum-parsimony tree generated based on the 16S rRNA gene sequences of strain ProA8^{T} and related reference taxa. The numbers at the branching nodes indicate the percentage of 1,000 bootstrap replications (only values >70% are shown). GenBank accession numbers for 16S rRNA gene sequences are provided in parentheses. The scale bar corresponds to 10 substitutions per nucleotide position**.** *Agrococcus jenensis* DSM 9580^{T} was used as an out-group.

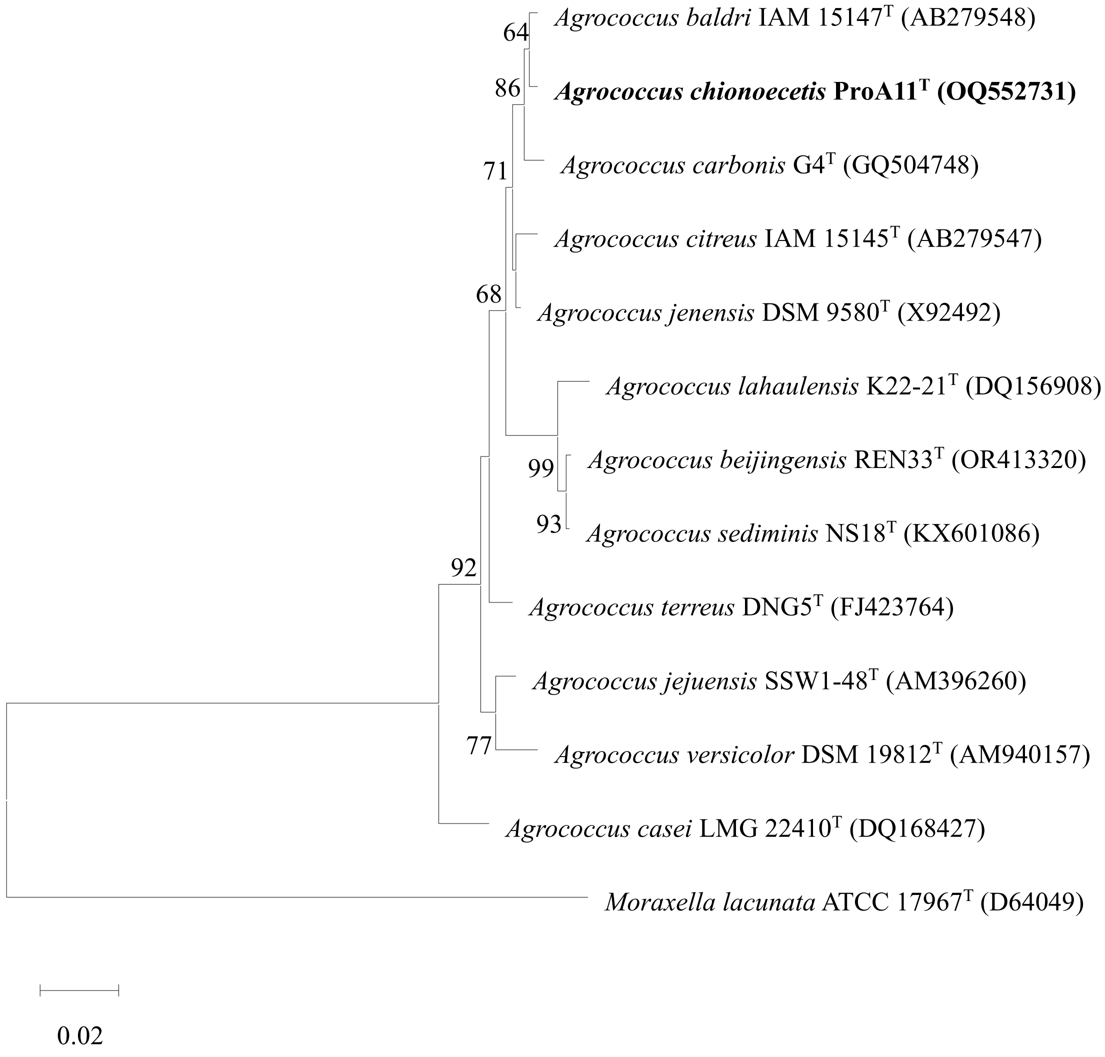

Fig. S3. Neighbor-joining tree generated based on the 16S rRNA gene sequences of strain ProA11^{T} and related reference taxa. The numbers at the branching nodes indicate the percentage of 1,000 bootstrap replications (only values >70% are shown). GenBank accession numbers for 16S rRNA gene sequences are provided in parentheses. The scale bar corresponds to 0.02 substitutions per nucleotide position**.** *Moraxella lacunata* ATCC 17967^{T} was used as an out-group.

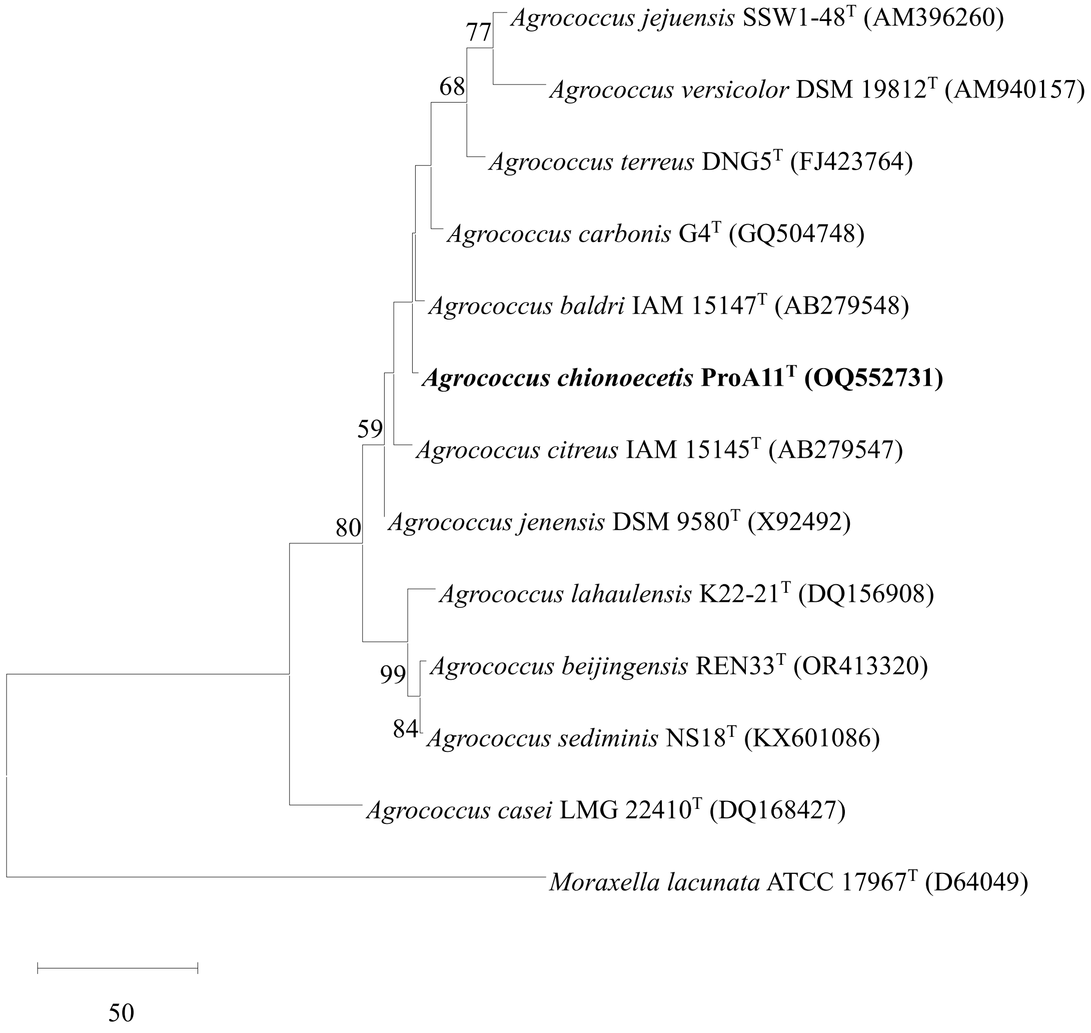

Fig. S4. Maximum-parsimony tree generated based on the 16S rRNA gene sequences of strain ProA11^{T} and related reference taxa. The numbers at the branching nodes indicate the percentage of 1,000 bootstrap replications (only values >70% are shown). GenBank accession numbers for 16S rRNA gene sequences are provided in parentheses. The scale bar corresponds to 50 substitutions per nucleotide position**.** *Moraxella lacunata* ATCC 17967^{T} was used as an out-group.

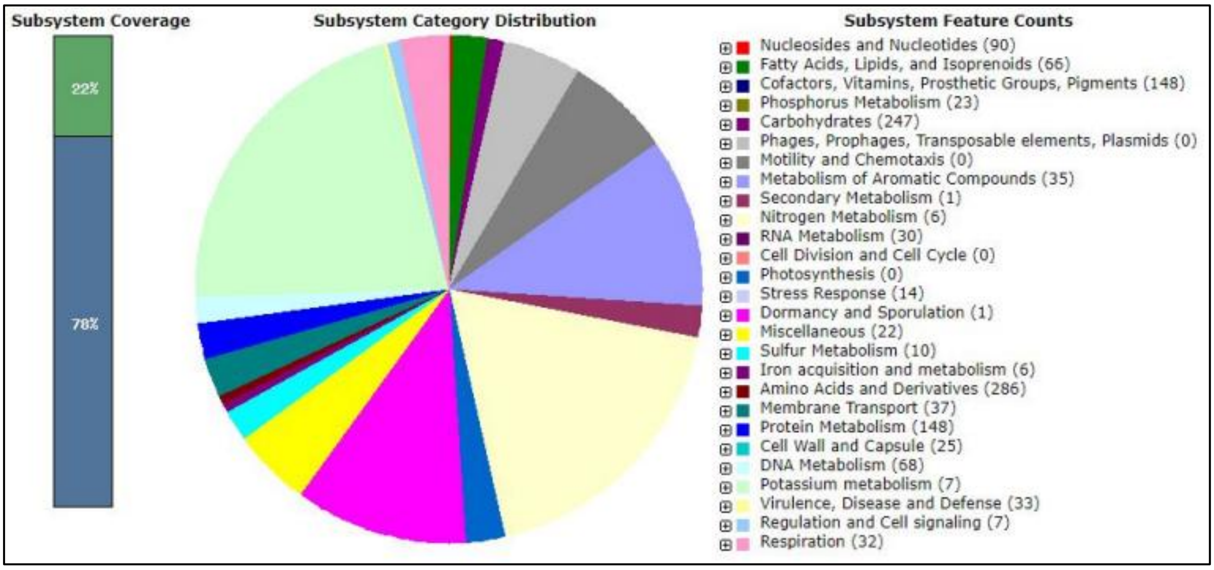

Fig. S5. Genome annotation of strain ProA8^T conducted by RAST (Rapid Annotation using Subsystem Technology) server.

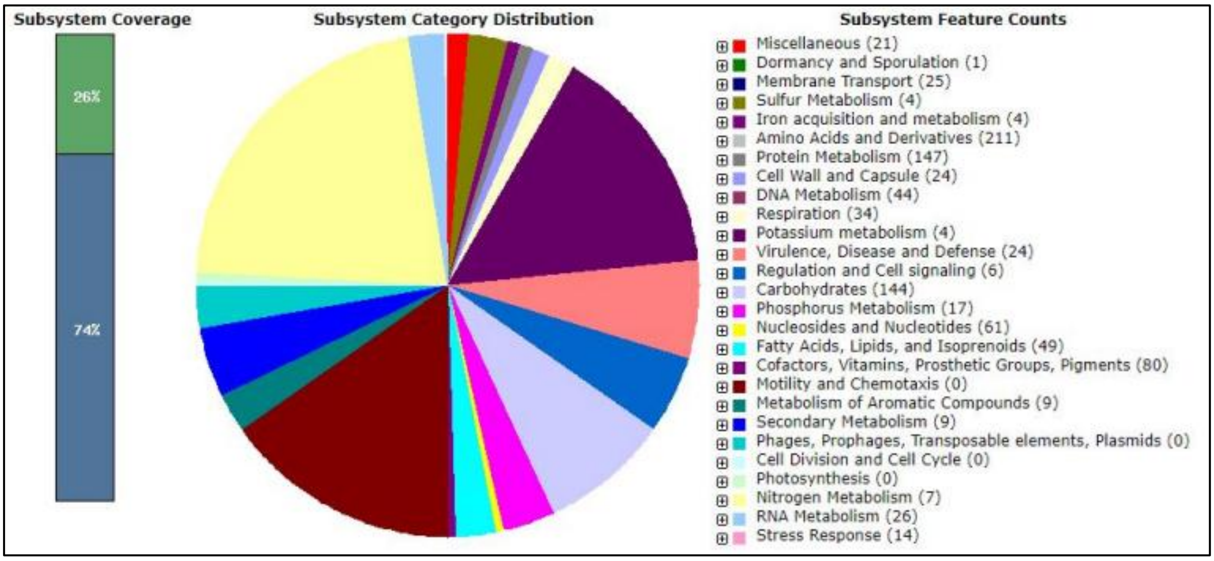

Fig. S6. Genome annotation of strain ProA11^{T} conducted by RAST (Rapid Annotation using Subsystem Technology) server.

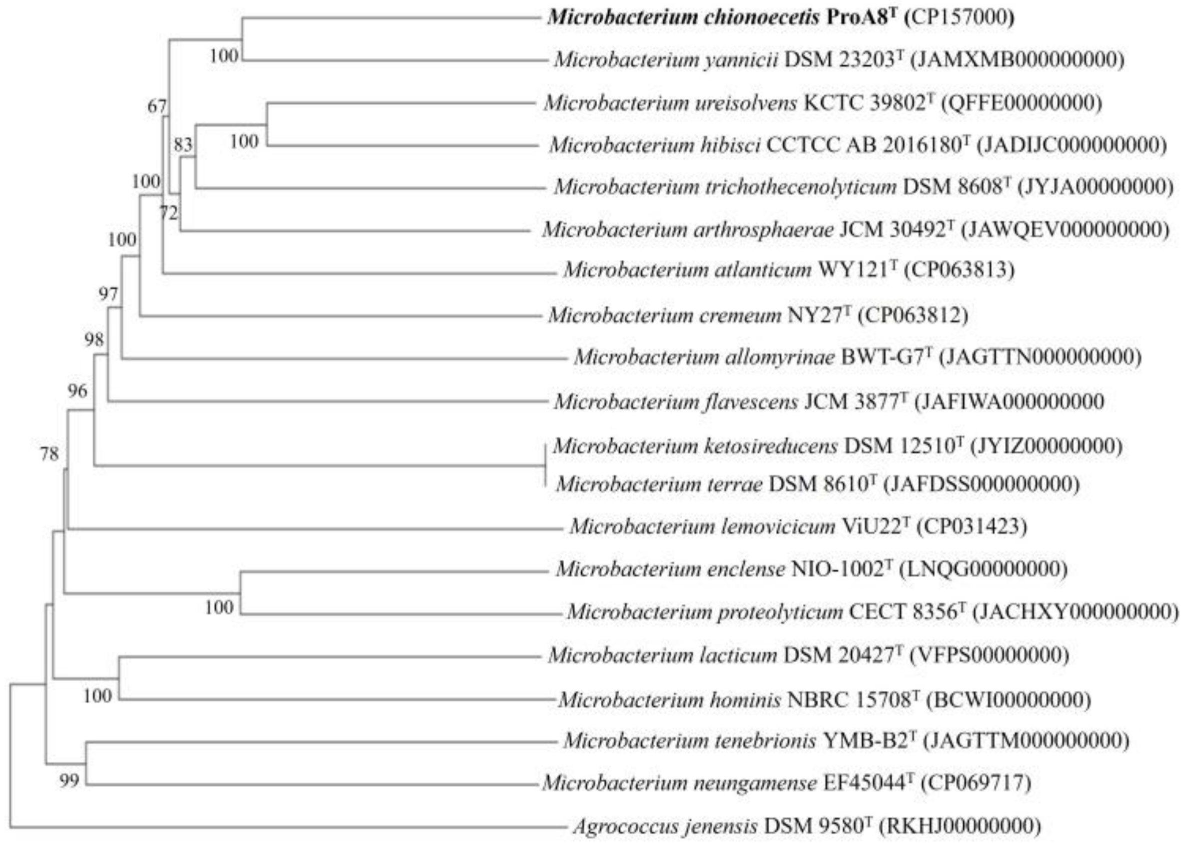

Fig. S7. Phylogenomic tree generated with FastME 2.1.6.1 based on GBDP distances computed from genome data of strain ProA8^{T} and reference strains. The numbers illustrated at the branches are GBDP pseudo-bootstrap support values from 100 replications.

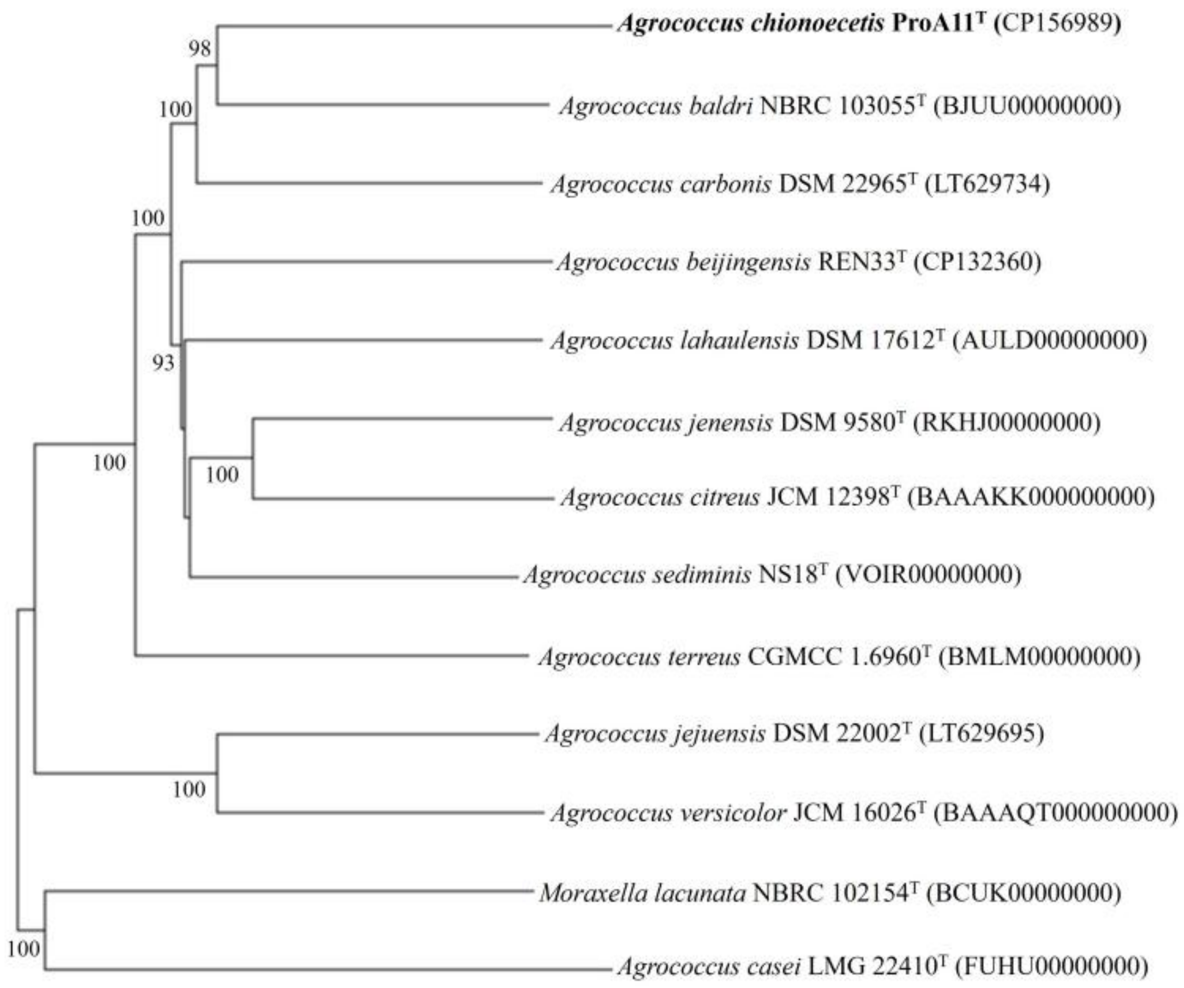

Fig. S8. Phylogenomic tree generated with FastME 2.1.6.1 based on GBDP distances computed from genome data of strain ProA11^{T} and reference strains. The numbers illustrated at the branches are GBDP pseudo-bootstrap support values from 100 replications.

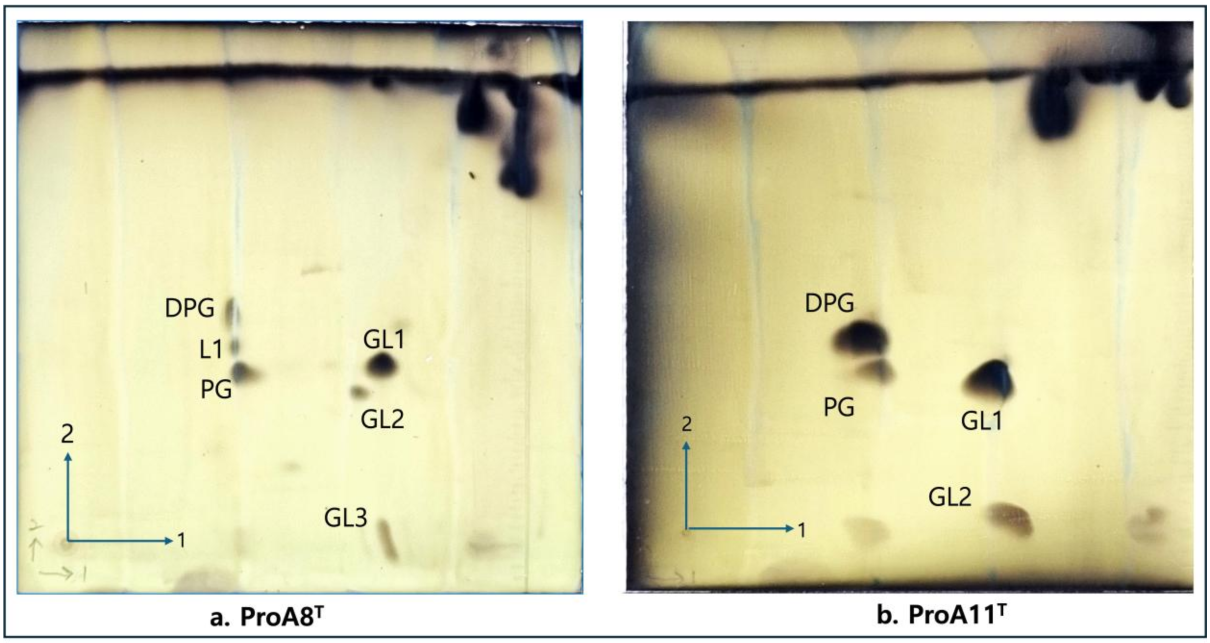

Fig. S9. Thin-layer chromatograms of the polar lipids from strains ProA8^T and ProA11^T. Abbreviations: DPG, Diphosphatidylglycerol; PG, Phosphatidylglycerol; unidentified glycolipids (GL1-GL3), and unidentified polar lipids (L1).
